# Supplementary material for: Vegetation and Environmental Changes on Contaminated Soil Formed on Waste from an Historic Zn-Pb Ore-Washing Plant
Source: Biology (Basel). 2021 Nov 27;10(12):1242. doi: 10.3390/biology10121242 (PMC8698733; doi:10.3390/biology10121242)
Supplement: Supplementary file 1 [file biology-10-01242-s001.zip › biology-1478942-supplementary.pdf]

Table S1. Share of families, genera and life forms in the flora of Zakawie

| **Family** | **Genera** | **Number of species in genera** | **Name of species** | **Period of observations** | | **Raunkiar`s life forms** | |
|---|---|---|---|---|---|---|---|
| | | | | 1999 | 2019 | 2009 | 2019 |
| *Poacea* | *Arrhenatherum* | 1 | Arrhenatherum elatius (L.) P.Beauv. ex J.Presl & C.Presl. | 1 | 1 | H | H |
| *Rosaeceae* | *Agrimonia* | 1 | Agrimonia eupatoria L. | 0 | 1 | | H |
| *Plumbaginaceae* | *Armeria* | 1 | Armeria maritima subsp. halleri (Wallr.) Rothm. | 1 | 1 | H | H |
| *Ranunculaceae* | *Ranunculus* | 1 | Batrachium aquatile Dumort. | 1 | 0 | Hy, T | |
| *Betulaceae* | *Betula* | 1 | Betula pendula Roth. | 0 | 1 | | M |
| *Ranunculaceae* | *Caltha* | 1 | Caltha palustris L. | 1 | 1 | Hy | Hy |
| *Compositae* | *Carduus* | 1 | Carduus nutans L. | 0 | 1 | | H |
| *Cyperaceae* | *Carex* | 6 | Carex canescens L. | 0 | 1 | | H |
| *Cyperaceae* | *Carex* | | Carex gracilis Curtis | 1 | 1 | G,H | G,H |
| *Cyperaceae* | *Carex* | | Carex paniculata L. | 0 | 1 | | H |
| *Cyperaceae* | *Carex* | | Carex remota L. | 0 | 1 | | H |
| *Cyperaceae* | *Carex* | | Carex riparia Curtis | 1 | 1 | Hy, H | Hy, H |
| *Cyperaceae* | *Carex* | | Carex rostrata Stokes | 1 | 0 | Hy, H | |
| *Compositae* | *Centaurea* | 2 | Centaurea scabiosa L. | 1 | 1 | H | H |
| *Compositae* | *Centaurea* | | Centaurea stoebe Tausch | 1 | 1 | H | H |
| *Compositae* | Cirsium | 2 | Cirsium oleraceum (L.) Scop. | 0 | 1 | | H |
| *Compositae* | Cirsium | | Cirsium rivulare | 1 | 1 | H | H |
| *Cornaceae* | Cornus | 1 | Cornus sanguinea L. | 0 | 1 | | N |
| *Betulaceae* | Corylus | 1 | Corylus avellana L. | 0 | 1 | | N |
| *Poacea* | Corynephorus | 1 | Corynephorus canescens (L.) P.Beauv. | 1 | 0 | H | |
| *Orchidaceae* | Dactylorhiza | 1 | Dactylorhiza maculata (L.) Soó | 1 | 0 | G | |
| *Poacea* | Deschampsia | | Deschampsia caespitosa (L.) P.Beauv. | 0 | 1 | | H |
| Caryophyllaceae | Dianthus | 2 | Dianthus deltoides L. | 1 | 1 | C, H | C, H |
| Caryophyllaceae | Dianthus | | Dianthus carthusianorum L. | 0 | 1 | | C |
| *Orchidaceae* | Epipactis | 1 | Epipactis palustris (L.) Crantz | 1 | 0 | G | |
| Equisetaceae | Equisetum | 1 | Equisetum palustre L. | 0 | 1 | | G |
| Celastraceae | Euonymus | 1 | Euonymus europaeus L. | 0 | 1 | | N |
| Compositae | Eupatorium | 1 | Eupatorium cannabinum L. | 0 | 1 | | H |
| Euphorbiaceae | Euphorbia | 1 | Euphorbia esula L. | 1 | 1 | H | H |
| Apiaceae | Falcaria | 1 | Falcaria vulgaris Bernh. | 1 | 1 | H | H |
| *Poacea* | Festuca | 1 | Festuca ovina L. | 1 | 1 | H | H |
| Rhamnaceae | Frangula | 1 | Frangula alnus Mill. | 1 | 1 | N | N |
| Rubiacea | Galium | | Galum verum L. | 0 | 1 | | H |
| Gentianaceae | Gentiana | 1 | Gentiana pneumonanthe L. | 1 | 0 | H | |
| Geraniaceae | Geranium | 1 | Geranium pratense L. | 0 | 1 | | H |
| *Rosaeceae* | Geum | | Geum rivale L. | 0 | 1 | | H |
| Iridaceae | Gladiolus | | Gladiolus imbricatus L. | 1 | 0 | G | |
| Compositae | Hieracium | | Hieracium pilosella L. | 1 | 0 | H | |
| Iridaceae | Iris | 1 | Iris sibirica L. | 1 | 0 | G | |
| Campanulaceae | Jasione | 1 | Jasione montana L. | 1 | 0 | H | |
| Juncaceae | Juncus | 2 | Juncus conglomeratus L. | 0 | 1 | | H |
| Juncaceae | Juncus | | Juncus effusus L. | 0 | 1 | | H |
| *Poacea* | Koeleria | 1 | Koeleria glauca (Spreng.) DC. | 1 | 0 | H | |
| *Orchidaceae* | Listera | 1 | Listera ovata (L.) R.Br. | 0 | 1 | G | |
| Araceae | Lemna | 1 | Lemna minor L. | 1 | 0 | Hy | |
| Apiaceae | Libanotis | 1 | Libanotis pyrenaica Bourg. | 1 | 1 | H | H |
| Lamiaceae | Lycopus | 1 | Lycopus europaeus L. | 0 | 1 | | H, Hy |
| Lamiaceae | Mentha | 2 | Mentha aquatica L. | 1 | 1 | H, Hy | H, Hy |
| Lamiaceae | Mentha | | Mentha x piperita L. | 0 | 1 | H, Hy | |
| *Poacea* | Molinia | | Molinia caerulea (L.) Moench | 1 | 1 | H | H |
| Ophioglossaceae | Ophioglossum | | Ophioglossum vulgatum | 1 | 0 | G | |
| Asparagaceae | Ornithogalum | | Ornithogalum umbellatum L. | 0 | 1 | | G |
| Rosaeceae | Padus | 2 | Padus padus L. | 0 | 1 | | M |
| Rosaeceae | Padus | | Padus serotina Ehrh. | 0 | 1 | | N |
| Pinaceae | Pinus | 1 | Pinus sylvestris L. | 1 | 1 | M | M |
| Plantaginaceae | Plantago | 3 | Plantago lanceolata L. | 1 | 1 | H | H |
| Plantaginaceae | Plantago | | Plantago major L. | 1 | 1 | H | H |
| Plantaginaceae | Plantago | | Plantago media L. | 1 | 1 | H | H |
| *Poacea* | Poa | 1 | Poa annua L. | 1 | 1 | H, T | H,T |
| Salicaceae | Populus | 1 | Populus tremula L. | 0 | 1 | | M |

| | | | | | | | |
|---|---|---|---|---|---|---|---|
| *Rosaeceae* | Potentilla | | Potentilla neumanniana Rchb. | 1 | 0 | H | |
| *Rosaeceae* | Prunus | 1 | Prunus spinosa L. | 1 | 1 | N | N |
| Rhamnaceae | Rhamnus | 1 | Rhamnus cathartica L. | 1 | 1 | N | N |
| *Rosaeceae* | Rosa | 1 | Rosa canina L. | 0 | 1 | | N, li |
| Polygonaceae | Rumex | 1 | Rumex acetosella | 1 | 0 | G, H, T | |
| Salicaceae | Salix | 2 | Salix caprea L. | 1 | 1 | N,M | N,M |
| Salicaceae | Salix | | Salix cinerea L. | 1 | 1 | N | N |
| Adoxaceae | Sambucus | 1 | Sambucus nigra L. | 1 | 1 | N | N |
| *Rosaeceae* | Sanquisorba | 1 | Sanguisorba minor Scop. | 0 | 1 | | H |
| Caprifoliaceae | Scabiosa | 1 | Scabiosa ochroleuca L. | 1 | 1 | H | H |
| Caryophyllaceae | Scleranthus | 1 | Scleranthus annuus L. | 1 | 0 | T | |
| Crassulaceae | Sedum | 1 | Sedum acre L. | 1 | 0 | C | |
| Typhaceae | Sparganium | 1 | Sparganium erectum L. | 1 | 1 | Hy | Hy |
| Compositae | Solidago | 1 | *Solidago canadensis* L. | 0 | 1 | | G,H |
| Lamiaceae | Thymus | 1 | *Thymus pulegioides* L. | 1 | 1 | C | C |
| Caprifoliaceae | Valeriana | 1 | *Valeriana officinalis* L. | 0 | 1 | | H |
| Adoxaceae | Viburnum | 1 | *Viburnum opulus* L. | 0 | 1 | | N |
| | | | | 46 | 60 | | |
| | | | | | | | |
| | | | | % | % | | |
| | | | **EXPLANATIONS:** | 1999 | 2019 | | |
| | | | M - megaphanerophyte | 3,5 | 6,3 | | |
| | | | N - nanophanerophyte | 10,5 | 18,8 | | |
| | | | C - herbaceous chamaephyte | 5,3 | 4,7 | | |
| | | | H - hemicryptophyte | 45,6 | 56,3 | | |
| | | | G - geophyte | 14,0 | 6,3 | | |
| | | | T - therophyte | 7,0 | 0,0 | | |
| | | | H - hydrophyte | 14,0 | 7,8 | | |
